# Mesoporous Materials for Drug Delivery and Theranostics

**DOI:** 10.3390/pharmaceutics12111108

**Published:** 2020-11-18

**Authors:** Valentina Cauda, Giancarlo Canavese

**Affiliations:** Department of Applied Science & Technology, Politecnico di Torino, Corso Duca degli Abruzzi 24, 10129 Turin, Italy

Mesoporous materials, especially those made of silica or silicon, are capturing great interest in the field of nanomedicine. Thanks to their exceptional porous structure and surface area, their homogeneous and tunable pore size, the ease of surface functionalization, the capability to establish host–guest interactions with other molecules protecting them from the external environment, and finally their biocompatibility, mesoporous materials enable a broad series of biologically relevant interventions and interactions with cells. The deep investigation on mesoporous nanoparticles has contributed to develop smart and stimuli-responsive nanotools for controlled drug- or gene-delivery and with imaging capabilities.

This Special Issue of Pharmaceutics is therefore dedicated to the most recent advances in the use of mesoporous nanostructures in the field of theranostis, specifically for cancer therapy, and in advanced tissue engineering.

To have a proper overview in the specific field of mesoporous silica materials for drug delivery, targeting, and theranostics applications, the review from Prof. Maria Vallet-Regì and coworkers is very relevant [1]. Here, the authors analyze various strategies about the encapsulation and delivery of macromolecules of biological interests (i.e., enzymes, therapeutic or antibacterial proteins, growth factors, therapeutic or antibacterial peptides, glycan-based macromolecules, and nucleic acids for gene modulation and silencing, like miRNA, siRNA, and DNA). The relevant figures of merit for the correct design of mesoporous silica nanoparticles (MSNs), such as pore size and shape, nanoparticle dimensions, surface chemistry, and colloidal stability, are considered. Furthermore, the location of the biomacromolecules (either at the external surface or in the mesopores) and the bond types to the silica surface (relying on physical adsorption or chemical grafting with various chemical and sometimes triggerable bonds) are reviewed.

In the review of Dr. Sugata Barui et al. [2], a special focus is given to both the surface decoration of MSNs by ligands for active targeting of cancer cells, exploiting overexpressed receptors, and to the use of stimuli-responsive gatekeepers for the controlled release of drugs to the disease site, avoiding leakage to healthy tissues. In addition, the multimodal modifications of the MSNs for simultaneous active targeting and stimuli-responsive behavior are reviewed with the most recent applications in vitro and in vivo. Applications of MSNs in cancer diagnosis and finally in theranostics are also proposed.

Experimental results on the most recent advances in nucleic acid delivery and efficient cancer cell targeting are provided in the work of Prof. Thomas Bein and coworkers [3]. Here, multiple core-shell functionalized MSNs were used to exploit a positively charged pore surface for miRNA loading and protection of this fragile cargo in the nanoparticle interior. On the outer surface, a block copolymer was electrostatically bound with the purpose of pore gatekeeping and endosomal release triggering. Finally, a targeting peptide GE11 for the epidermal growth factor receptor (EGFR) was used to enhance the MSN uptake in bladder cancer cells in vitro and provide the miRNA delivery for gene knockdown.

Despite silica-based mesoporous materials, this Special Issue also provides recent and prominent insights in the use of mesoporous bioactive glasses (MBGs) for tissue engineering applications.

Specifically, for bone tissue engineering applications, the work of Profs. Ying Wan and Jiliang Wu [4] shows that MBGs in the form of nanoparticles can be used as carriers for insulin-like growth factor-1 and can be efficiently incorporated into an injectable hydrogel matrix. Interestingly, the sol to gel transition of the hydrogel is engineered such that it can happen at physiological temperature and pH, resulting in gel with high porosity and interconnected pores, which is thus suitable for sustaining the delivery of the cargo over weeks and maintaining its biological functions, as proven by in vitro tests with osteoblasts.

Also representative is the work of Boffito et al. [5], where MBG incorporated into a hydrogel matrix was successfully designed to simultaneously release both copper ions, with pro-angiogenic and anti-bacterial effects, and an anti-inflammatory drug. The work aims to propose a multifunctional platform for tissue healing, in particular bone healing, where, on the one hand, the thermosensitive hydrogel concentrates and maintains the MBG carriers at the diseased site and, on the other hand, the in situ and prolonged co-release of ions and drugs is achieved.

Top-down fabricated mesoporous-based nanomaterials are also presented in this Special Issue. In the work of Prof. Alessandro Grattoni and coworkers [6], a silicon nanofluidic membrane incorporating gate electrodes is presented. This nanochannel-based device is able to modulate the transport of charged molecules, here in particular methotrexate, used to treat rheumatoid arthritis, and quantum dots, which are useful for bio-imaging applications. The electrostatic gated nanochannel permeability was proven to deliver the cargos at low applied voltages in vitro, modulating the transport release performances.

In the work of Profs. Natalia Malara, Francesco Gentile, and coworkers [7], mesoporous silicon structures coated with gold nanoparticles are fabricated, allowing a high level of control over the surface at the nanoscale. These excellent characteristics enable the device to be used for theranostics purposes (i.e., first supporting the growth and proliferation of cancer cells over the nanomaterial surface, then allowing an antitumor drug uptake and subsequent delivery against cancer cells thanks to the mesopores, and finally providing imaging of the biological system by surface enhanced Raman spectroscopy (SERS) due to the presence of the gold nanoparticles).

The review of Dr. Tania Limongi, Francesca Susa, and coworkers [8] presents an innovative perspective highlighting the synthetic approaches, characteristics, and roles of 3D-printed mesoporous materials as customizable and personalized scaffolds for drug delivery studies and tissue engineering applications. Such 3D-printed mesoporous materials can provide not only a solid support for cell growth in a 3D fashion, but also and most importantly can be ad-hoc designed and customized for personalized therapy to patients, or for realistic in vitro drug delivery studies, or finally for assisting cell growth for a tissue-specific model.

As a concluding remark, with this Special Issue, we hope we have contributed to highlighting the role of mesoporous materials in cancer cell theranostics and tissue engineering, providing insights from their synthesis, surface functionalization, and characterization up to their smart and stimuli-responsive behavior with customizable properties for advanced and personalized biomedical applications.

## References

[1] Castillo R.R., Lozano D., Vallet-Regí M. (2020). Mesoporous Silica Nanoparticles as Carriers for Therapeutic Biomolecules. Pharmaceutics.

[2] Barui S., Cauda V. (2020). Multimodal Decorations of Mesoporous Silica Nanoparticles for Improved Cancer Therapy. Pharmaceutics.

[3] Haddick L., Zhang W., Reinhard S., Möller K., Engelke H., Wagner E., Bein T. (2020). Particle-Size-Dependent Delivery of Antitumoral miRNA Using Targeted Mesoporous Silica Nanoparticles. Pharmaceutics.

[4] Min Q., Yu X., Liu J., Zhang Y., Wan Y., Wu J. (2020). Controlled Delivery of Insulin-like Growth Factor-1 from Bioactive Glass-Incorporated Alginate-Poloxamer/Silk Fibroin Hydrogels. Pharmaceutics.

[5] Boffito M., Pontremoli C., Fiorilli S., Laurano R., Ciardelli G., Vitale-Brovarone C. (2019). Injectable Thermosensitive Formulation Based on Polyurethane Hydrogel/Mesoporous Glasses for Sustained Co-Delivery of Functional Ions and Drugs. Pharmaceutics.

[6] Di Trani N., Silvestri A., Wang Y., Demarchi D., Liu X., Grattoni A. (2020). Silicon Nanofluidic Membrane for Electrostatic Control of Drugs and Analytes Elution. Pharmaceutics.

[7] Coluccio M.L., Onesto V., Marinaro G., Dell’Apa M., De Vitis S., Imbrogno A., Tirinato L., Perozziello G., Di Fabrizio E., Candeloro P. (2020). Cell Theranostics on Mesoporous Silicon Substrates. Pharmaceutics.

[8] Limongi T., Susa F., Allione M., di Fabrizio E. (2020). Drug Delivery Applications of Three-Dimensional Printed (3DP) Mesoporous Scaffolds. Pharmaceutics.

